# Bond Strength of Composite CFRP Reinforcing Bars in Timber

**DOI:** 10.3390/ma8074034

**Published:** 2015-07-03

**Authors:** Marco Corradi, Luca Righetti, Antonio Borri

**Affiliations:** 1 Department of Mechanical and Construction Engineering, Northumbria University, 212 Wynne-Jones Building, Newcastle upon Tyne NE1 8ST, UK; 2 Department of Mechanical and Construction Engineering, Northumbria University, 209 Wynne-Jones Building, Newcastle upon Tyne NE1 8ST, UK; E-Mail: luca.righetti@northumbria.ac.uk; 3 Department of Engineering, University of Perugia, 92 Via Duranti, Perugia 06125, Italy; E-Mail: antonio.borri@unipg.it

**Keywords:** timber, composite materials, carbon fibre, epoxy resin, bonding, testing

## Abstract

The use of near-surface mounted (NSM) fibre-reinforced polymer (FRP) bars is an interesting method for increasing the shear and flexural strength of existing timber members. This article examines the behaviour of carbon FRP (CFRP) bars in timber under direct pull-out conditions. The objective of this experimental program is to investigate the bond strength between composite bars and timber: bars were epoxied into small notches made into chestnut and fir wood members using a commercially-available epoxy system. Bonded lengths varied from 150 to 300 mm. Failure modes, stress and strain distributions and the bond strength of CFRP bars have been evaluated and discussed. The pull-out capacity in NSM CFRP bars at the onset of debonding increased with bonded length up to a length of 250 mm. While CFRP bar’s pull-out was achieved only for specimens with bonded lengths of 150 and 200 mm, bar tensile failure was mainly recorded for bonded lengths of 250 and 300 mm.

## 1. Introduction

With increasing focus on the development of sustainable construction systems, the reinforcement of existing wood members and the use of timber in new constructions is at present receiving much attention.

In order to upsurge the useful life of wood structural elements, it is necessary to afford suitable retrofitting techniques. Timber, when used as a structural material, is constantly exposed to several agents of deterioration (insects assault, moisture variation, aging, biological attack, *etc*.), which reduce the strength and stiffness, and reinforcement interventions are often necessary to increase the capacity or to reduce flexural deflections. Many innovative techniques are available in the literature, which consider the use of traditional materials, such aluminium and steel rods, composite materials, such as carbon and glass fibre-reinforced polymers (FRPs), and, more recently, natural-based composite materials, such basalt FRP. In many cases these reinforcements are in the form of rods or bars.

In the first studies, the use of steel rods [1,2] glued on glulam beams produced interesting increases in capacity and stiffness. Recent research programs involving the reinforcement of wood beams have also examined the use of FRP bars applied on the tension side. FRP materials have excellent mechanical properties and exhibit very good characteristics in relation to long-term behaviour [3,4]. FRPs have been employed either to improve flexural and shear characteristics of existing structures or to reduce the dimension of new timber structures. Currently, the restricted data are accessible on the bond behaviour of FRP rods in timber, and design guidelines provided in Eurocodes and national standards for steel bars cannot be properly used for this purpose due to essential differences in surfaces deformations and mechanical properties.

Bars are usually glued into grooves realized along the direction parallel to the beams’ fibres using epoxy resins. The use of near-surface mounted (NSM) FRP bars as a replacement for steel has been encouraged, because it implicates higher mechanical properties, ease of application, a high stiffness-to-weight ratio (10- to 15-times higher than the steel) and better long-term behaviour. Several studies [3,4] have been carried out in order to analyse the mechanical characteristics of FRP bars, in particular their tensile stress, Young’s modulus, ultimate strain and creep behaviour, and results confirmed the good properties of the material that could constitute a suitable solution instead of the steel bar to strengthen concrete [5,6,7], masonry [8,9] and timber members [10,11,12,13].

Thus, current research on wood reinforcement has focused on the use of FRP strips or bars epoxy bonded to wood solid or glulam beams. However the response of the interface bond timber-epoxy-bar under loading is not yet fully defined, and additional information is needed to develop specifications and design values for reinforcement of timber with FRP. Gentile *et al*. [14] carried out several bending tests on a large number of half-scale timber beams reinforced with different diameter glass fibre-reinforced polymer (GFRP) bars, bonded with epoxy resin inside grooves realized in different position on the tensile surface. The results showed an increase of ultimate strength between 18% and 46% in the reinforced beams. Borri *et al*. [15] used carbon FRP (CFRP) bars, applied on the tensile side using epoxy resins, to reinforce solid timber beams, which produced an increase of the capacity up to 52%. GFRP and CFRP rods have been also used, with encouraging results, also for the reinforcement of glulam beams [16,17,18,19,20].

However, the increasing production costs of FRPs are significantly widening the field of research, especially toward natural materials, which readily available and considerably more economical. Recently, Raftery *et al*. [21] tested bending timber beams strengthened with basalt fibre-reinforced polymer (BFRP) bars bonded in notches realized on the tensile zone of Irish spruce beams exhibiting an ultimate capacity over 23% of the unreinforced beams. Several pull-out tests were carried out to investigate the bond capacity of FRP bars glued in timber specimens [22,23,24,25], in particular used for the connection of timber element. The results showed that the main failure mode was longitudinal splitting and pull-out of the rods along with a timber volume surrounding the bonded length; however, capacity increases with the growth of bonded length and notched size.

The above cited experimental results show that a debonding failure of the FRP may occur in some cases because of the push-off of the split timber near the beam midspan. However, it is unclear if this depends on the grade of the timber material, on the type of resin used to apply the FRP bars or on the position of the bar reinforcement. From recent studies on the behaviour of bonded FRP bars to solid and glulam wood, it is apparent that the problem is quite complicated, both experimentally and analytically, and more experimental data are necessary to address the problem. The bond behaviour of different wood species is another aspect to consider: in Northern Europe, the common use of faster-grown wood species, mainly softwood (fir, larch, *etc*.), which produce lower grade timber, can particularly benefit from the reinforcement with FRP bars, but it may determine problems at the timber-epoxy interface, compared to the use of hardwood (oak, chestnut, *etc*.).

This article examines the behaviour of carbon FRP bars in soft (fir) and hardwood (chestnut) under direct pull-out conditions. Results were previously partially presented in [26]. The objective of this experimental program is to investigate the bond strength between composite bars and timber: bars were epoxied into small notches made into chestnut and fir wood members using a commercially-available epoxy system with bonded lengths varying from 150 to 300 mm.

**Figure 1:**
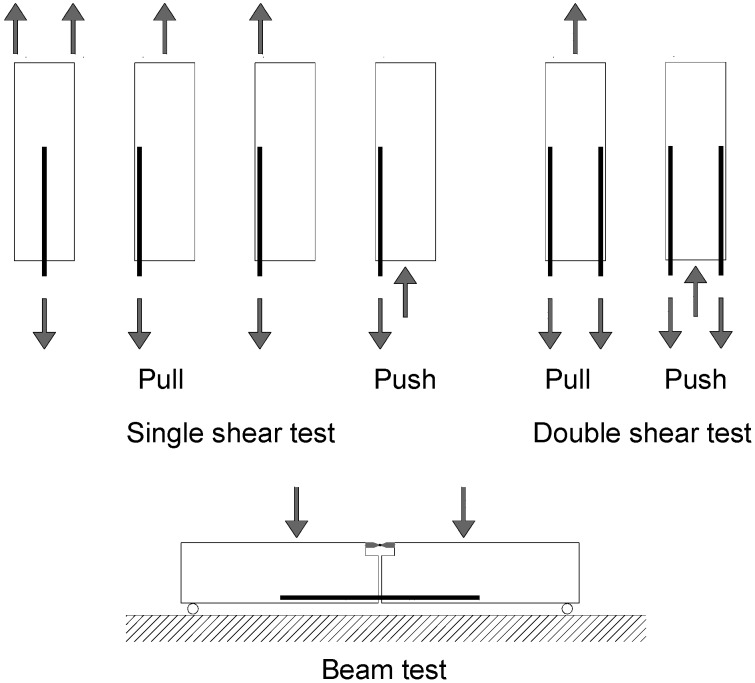
Different test setups.

There are many different experimental setups for determining the bond behaviour of the FRP substrate, amongst which single shear tests, double shear pull and push tests and shear bending tests are the most common (Figure 1). Since FRP bars are usually applied for flexural strengthening of beams, bending creates a tension zone; the stresses in the bond line between FRP and timber are much more complex than when pure tension tests are used. Thus, bending bond tests are more likely to represent the actual conditions than the direct pull-out tests, but significant limitations are also present for this setup [27].

In this experimental work, the double shear push test has been used as a result of its simplicity. However, it must be pointed out that numerical and experimental investigations have demonstrated that different test setups can produce different results, and small variations in setup may have significant effects [28].

## 2. Materials

### 2.1. Timber 

**Figure 2:**
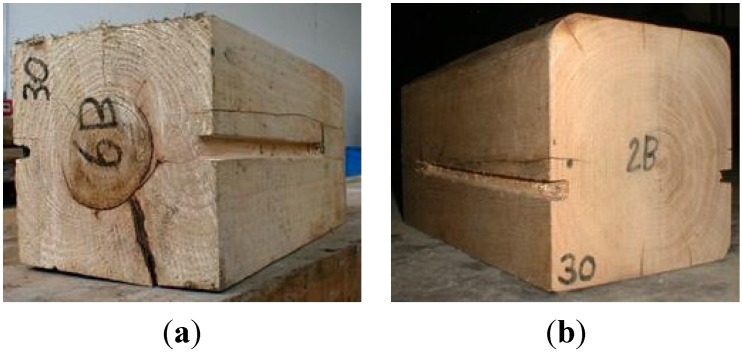
(**a**) Softwood prism (fir); (**b**) hardwood prism (chestnut).

Tests were carried out on prism specimens in fir (*Abies alba*) (Figure 2a) and chestnut wood (*Castanea sativa*) (Figure 2b). Specimen dimensions were 200 mm × 200 mm × 500 mm and 220 mm × 220 mm × 500 mm with a density of 453.6 and 448 kg/m^3^ for fir and chestnut wood, respectively. Moisture content was evaluated according to the EN 13183-1 standard [29]; the average value was 10.9% for fir and 17.12% for chestnut wood. Two notches, with cross-section of 14 mm × 15 mm, were realized into the side surfaces of the timber specimens, parallel to the grain, with a circular saw with different lengths (150, 200, 250 and 300 mm).

The timber material used in this experimental campaign was classified according to the EN 338 standard [30] in C24 and D24, according to [31], for fir and chestnut wood, respectively. In order to verify the quality of the timber, four-point-bending tests were carried out on four timber beams (two for each type of wood). The fir and chestnut wood bending strengths were 32.55 and 34 N/mm^2^, respectively. The limited number of characterization tests performed must be considered given the high natural variability of timber and the local character of the bonding tests.

### 2.2. CFRP Bars 

**Figure 3:**
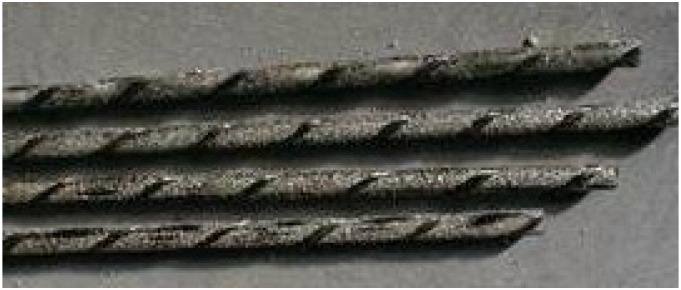
Carbon fibre-reinforced polymer (CFRP) bars: bars are sandblasted and superficially deformed.

Tests were performed to characterize the mechanical properties of the CFRP materials used in this investigation. CFRP unidirectional pultruded bars (Figure 3) were produced by MAC SpA (a product commercially known as “Leonardo”). NSM rods were 7.5 mm-diameter CFRP bars having a sandblasted surface to improve the bond characteristics and a deformed, helically-wound surface produced by fibre wraps. These rods are comparable to steel bars used in reinforced concrete in nominal dimensions; nevertheless, the main difference is the pultrusion process by which the bars are manufactured and the stress-strain behaviour. The pultrusion process produces bars with a reasonably constant cross-section. In the experimental campaign, bars with indentations were used; those were realized by wrapping a carbon fibre string around the bar before the resin dried. The stress-strain behaviour of CFRP bars is linear at all stress levels up to collapse, without showing any yielding behaviour.

The mechanical characterization of the CFRP bars was conducted on nine coupons tested in tension according to the ASTM D3039 standards [32], with a crosshead speed of 1 mm/min (displacement control mode). To avoid damaging the CFRP bars by the compression stresses introduced by the loading shoes of the test machine, the end of each specimen was inserted into a steel cylindrical pipe and fixed with epoxy resin. Test results are shown in Table 1.

**Table 1:** Mechanical properties of CFRP bars.

Nominal Diameter (mm)	Number of Specimens	Failure Load (kN)	Tensile Strength (N/mm^2^)	Strain at Failure (%)	Modulus of Elasticity *E*_b_ (N/mm^2^)
7.5	9	46.49 (1.82)	1053 (41.22)	0.69	151030

In parentheses: Standard deviation.

### 2.3. Epoxy System 

The epoxy system is constituted of two epoxy components: primer and saturant. Both of them are bi-component epoxy resins with a weight ratio of epoxy-to-curing agent of 3:1. The primer was initially applied on the wood surface to facilitate bonding. Notches were then filled with the saturant resin. Both components were mixed in ratio of 3:1 by volume and cured for 10 days at room temperature. The system was manufactured by MAC SpA. Mechanical characteristics of the saturant and primer were evaluated by testing 5 specimens in compression according to the ASTM D695 standard [33] and 5 specimens in tension according to the ASTM D638 standard [34]. Test results are shown in Table 2.

**Table 2:** Mechanical properties of the epoxy system.

	Saturant (N/mm^2^)	Primer (N/mm^2^)
Sample size	5	5
Compression strength	56.54	26.15
Sample size	5	5
Tensile strength	23.43	12.69
Modulus of elasticity	4510	426

**Figure 4:**
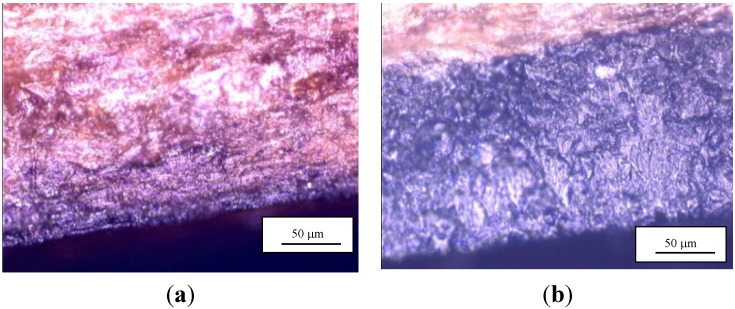
Microscopic analysis of the bonding: (**a**) primer-wood interface; (**b**) saturant-wood interface.

Microscopic analysis has been performed on chestnut wood samples. The primer and saturant were strained on the samples in order to analyse the penetration of the two resins into the wood. Microscopic analysis showed that the penetration of the primer in the chestnut wood was about 50 μm (Figure 4a), but that the penetration of the saturant was completely negligible (Figure 4b). This explains the necessity to use the epoxy primer, whose application was made by a brush, with a bond-line thickness varying between 0.02 and 0.06 mm [35].

## 3. Test Setup 

**Figure 5:**
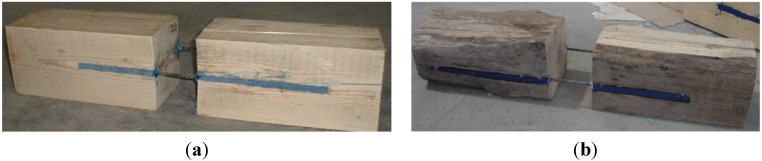
(**a**) Fir wood specimen; (**b**) chestnut wood specimen.

Twelve fir (Figure 5a) and twelve chestnut wood pull-out specimens (Figure 5b) have been obtained joining two square-section timber prisms with two unidirectional NSM CFRP bars positioned inside notches and secured in place using the epoxy system. The total number of pull-out specimens was 24. Fir and chestnut wood specimens are identified by the letters “SF” and “SC”, respectively.

**Figure 6:**
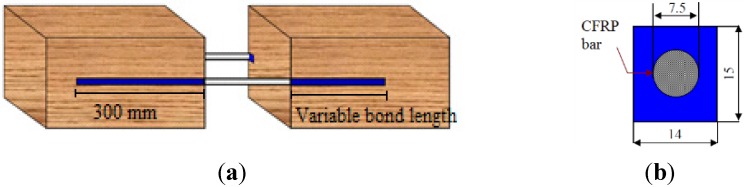
(**a**) CFRP bar schematic arrangement; (**b**) section of the notch (dimensions in mm).

Tests were divided into two categories. The first category of pull-out tests investigated the effect of different bonded lengths. Figure 6a shows the test schematic arrangement with bonded-in CFRP bars. Reinforcement bars were tested with bonded lengths of 150, 200, 250 and 300 mm. The pull-out specimens had a uniform rectangular notch size of 14 mm (width) × 15 mm (height) (Figure 6b). Based on the dimensions of the notch and the CFRP bar diameter, the glue-line thickness is approximately 3.5 mm: this value, relatively thick, was chosen to facilitate the penetration of the epoxy resin into the notch and to improve the bonding strength [35].

The curing time before testing was 15 days at room temperature. A mutual distance of 115 mm between the two prismatic elements was used to allow the allocation of the test equipment (hydraulic jack, steel plates).

## 4. Experimental Campaign 

Twenty-four specimens were tested to study the bonding behaviour of the CFRP bars with timber. The analysed parameters were the failure mode, maximum load (pull-out capacity), average bond stress on the lateral bar’s surface and on the interface between bar and epoxy resin. Furthermore, strain gauges were applied on the bar surface of four of the above specimens (SF_2, SF_4, SC_2 and SC_3), to evaluate the stress distribution along the CFRP bars.

**Figure 7:**
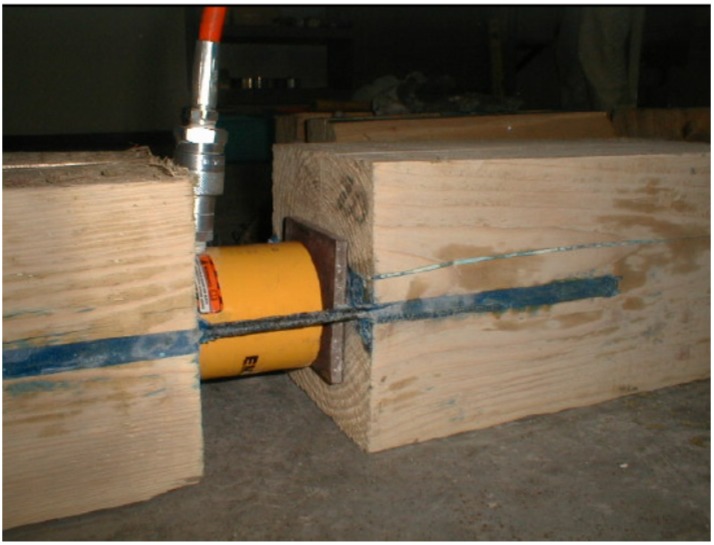
Pull-out test arrangement (tests without strain gauges).

Timber species and bonded length were the test variables considered in this work. Tests were carried out placing between the prismatic wood elements an Enerpac 20-ton hydraulic jack (Figure 7) with a stroke of 50 mm actuated by a 700-bar manual pump; the pushing cylinder of the jack was placed in contact with the timber prism surfaces. To avoid the crushing of the timber and shear along the grain, due to the application of the jack on a small surface, two square (110 mm × 110 mm) bearing steel plates were inserted between the jack and the timber specimen (Figure 7). While preventing timber local failure, the bearing plates had a clear distance from the notches to allow for timber failure near the epoxy-wood interface. The gradient of the pressure manually applied with the pump to the specimen was approximately 3 bar/s.

### 4.1. Tests without Strain Gauges 

The objective of these pull-out tests was to measure the bond strength for different bonded lengths. Twelve fir wood and twelve chestnut wood specimens were tested to investigate the behaviour of the CFRP bars epoxied into timber elements. Tensile stress σ in the bar could be computed from the externally-applied load using equilibrium. By assuming an equal distribution of the load between the CFRP bars and that shear stress was constant over the bar-epoxy interface, the bond strength τ_b_ of the single bar can be easily determined by dividing the poll-out load by the lateral bar surface.

Three different failure modes were recorded during the tests: (1) pull-out of the CFRP reinforcement from the epoxy substrate; (2) timber shear failure; and (3) tensile failure of the CFRP bars.

**Figure 8:**
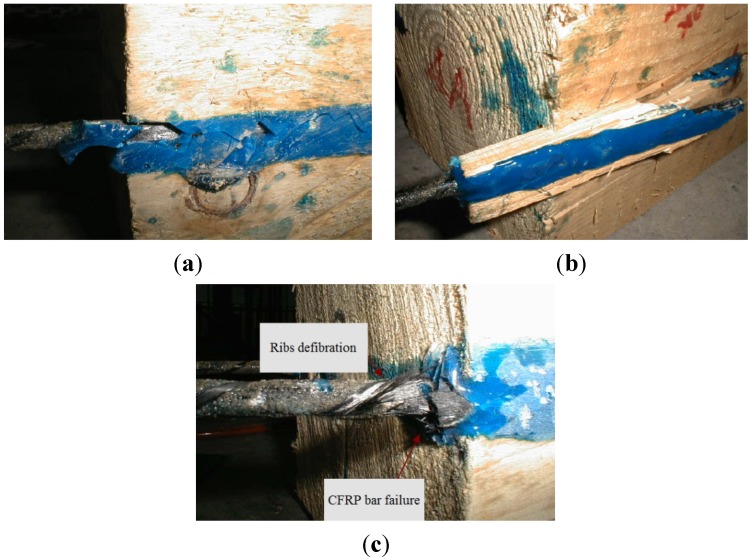
Failure modes: (**a**) bar pull-out; (**b**) timber shear failure; (**c**) CFRP bar tensile failure.

In detail, the first mode was characterized by the bar-epoxy interface failure and the subsequent pull-out of the CFRP bar from the epoxy resin (Figure 8a). This was the most frequent mode of failure for bonded lengths of 150 and 200 mm. The longitudinal micro-cracking that appeared was due to the compressive forces radiating out in an inclination that varies with rib surface. This cracking slowly propagated up with the increase of the load until a noticeable cracking of the epoxy resin and the subsequent slipping out of the bar occurred.

The second failure mode was frequent for soft wood (fir) specimens with small bonded lengths (150, 200 and 250 mm), and this mainly involved the wood material. Wood failure occurred when the failure surface was within the wood adjacent to the notch. After developing small shear cracks primarily located in the timber, specimens failed for the pull-out of the CFRP bar and a portion of the timber materials surrounding the bar (Figure 8b).

The third failure mode was characterized by the rupture of the FRP reinforcement (Figure 8c). The majority of pull-out specimens with a bonded length of 300 mm failed according to this mode. This failure was not instantaneous: the carbon fibres applied helicoidally over the CFRP bars failed first, followed by the tensile rupture of longitudinal carbon fibres.

As expected, the failure load increased with the bonded length. The average normal stress at failure σ, bond stresses at interface bar-resin τ_b_ and at interface resin-timber τ_ew_ were 530.3, 6.63 and 3.55 N/mm^2^ respectively, for fir wood specimens with a bonded length of 150 mm; similar results were measured for chestnut wood having the same bonded length (Table 3).

**Table 3:** Test results. SF, fir specimen; SC, chestnut specimen.

Index	Bonded Length (mm)	Maximum Load *F*_max_ (kN)	Tensile Normal Stress σ (N/mm^2^)	Bond Stress τ_b_ (N/mm^2^)	Bond Stress τ_ew_ (N/mm^2^)	Failure Mode
SF_1	150	23.96	542.6	6.78	3.63	Bar pull-out
SF_2	150	20.52	464.7	5.81	3.11	Timber shear failure
SF_7	150	25.77	583.6	7.30	3.90	Bar pull-out
		23.42 (2.67)	530.3 (60.4)	6.63 (0.75)	3.55 (0.40)	
SF_3	200	31.95	723.6	6.78	3.63	Timber shear failure
SF_4	200	33.78	765.0	7.17	3.84	Bar pull-out
SF_8	200	32.45	734.9	6.89	3.69	Timber shear failure
		32.73 (0.95)	741.2 (21.4)	6.95 (0.20)	3.72 (0.11)	
SF_5	250	47.23	1070	8.02	4.29	Timber shear failure
SF_6	250	47.92	1085	8.14	4.36	Bar tensile failure
SF_9	250	44.65	1011	7.58	4.06	Timber shear failure
		46.60 (1.72)	1055 (39.0)	7.92 (0.29)	4.24 (0.16)	
SF_10	300	48.08	1089	6.81	3.64	Bar tensile failure
SF_11	300	48.77	1104	6.90	3.69	Bar tensile failure
SF_12	300	44.95	1018	6.36	3.41	Timber shear failure
		47.27 (2.04)	1070 (46.1)	6.69 (0.29)	3.58 (0.15)	
SC_1	150	26.01	589.0	7.36	3.94	Timber shear failure
SC_2	150	26.74	605.6	7.57	4.05	Bar pull-out
SC_7	150	23.45	531.1	6.64	3.55	Bar pull-out
		25.40 (1.73)	575.2 (39.1)	7.19 (0.49)	3.85 (0.26)	
SC_3	200	28.7	650.0	6.09	3.26	Bar pull-out
SC_4	200	32.37	733.1	6.87	3.68	Bar pull-out
SC_8	200	32.12	727.4	6.82	3.65	Bar pull-out
		31.06 (2.05)	703.5 (46.4)	6.60 (0.44)	3.53 (0.23)	
SC_5	250	49.31	1117	8.38	4.48	Bar tensile failure
SC_6	250	47.91	1085	8.14	4.36	Bar tensile failure
SC_9	250	46.56	1054	7.91	4.23	Bar tensile failure
		47.93 (1.38)	1085 (31.1)	8.14 (0.23)	4.36 (0.13)	
SC_10	300	48.56	1100	6.87	3.68	Bar tensile failure
SC_11	300	49.56	1122	7.01	3.75	Bar tensile failure
SC_12	300	47.9	1085	6.78	3.63	Bar tensile failure
		48.67 (0.84)	1102 (18.9)	6.89 (0.12)	3.69 (0.06)	

In parentheses: Standard deviation.

By increasing the CFRP bar bonded length of 33% (from 150 to 200 mm), an almost consistent increment of the maximum load (pull-out capacity) and stresses was measured and calculated (31.1%). The average normal stress and bond stresses increased 39.8 and 22.3%, respectively, for softwood (fir) and hardwood (chestnut) specimens. Specimens with 200-mm bonded lengths mainly exhibited a failure due to pull-out of the bar from the notches.

**Figure 9:**
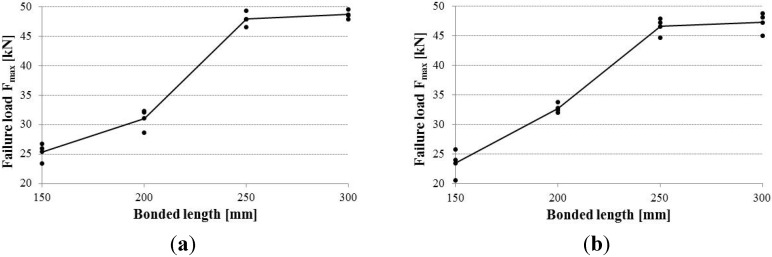
Failure load (pull-out capacity) *vs*. bonded length: (**a**) chestnut wood; (**b**) fir wood.

An interesting observation can be underlined for specimens with a longer bonded length. For both timber species, bar pull-out or timber shear failure modes were prevented when a bonded length of 250 mm was used. The bar’s failure appeared in correspondence to an average load *F*_max_ of 47.26 kN and an average bond stress at bar-epoxy interface of 8.03 N/mm^2^. The bonded length is the most influential parameter on the test; the differences between fir wood and chestnut wood specimens are very low for all the three different bonded length in terms of maximum load *F*_max_ (Figure 9), axial strength σ and bond strengths τ_b_ and τ_ew_.

Since the number of specimens tested was limited, results should be confirmed by a larger experimental programme. However, the emerging line seems quite correct: using bonded lengths greater than 250 mm does not cause an increase in the pull-out capacity in the CFRP bars. This was evident by comparing the results of specimens with a bonded length of 250 mm with the ones with 300 mm: fir wood specimens exhibited a capacity of 46.6 and 47.27 kN for 250 mm and 300 mm bonded lengths, respectively. For chestnut specimens, pull-out capacity increased from 47.93 to 48.67 kN.

### 4.2. Tests with Strain Gauges 

**Figure 10:**
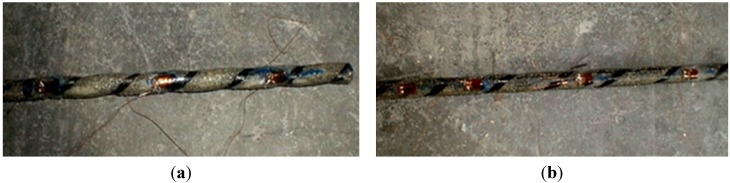
Strain gauge arrangement on FRP bar: (**a**) for a bonded length of 150 mm; (**b**) for a bonded length of 200 mm.

**Figure 11:**
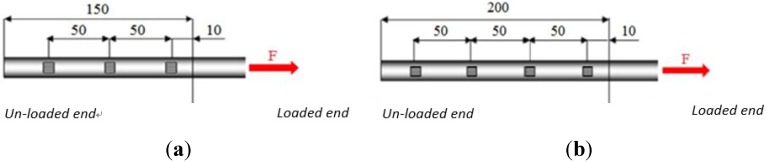
(**a**) Strain gauge arrangement along reinforcement (150-mm bonded length); (**b**) strain gauge arrangement along reinforcement (200-mm bonded length).

In order to evaluate the distribution of stress and strain, four pull-out tests [SF_2, SF_4, SC_2 and SC_3 (Table 3)] were carried out with the use of strain gauges. Strain gauges were produced by Micro-Measurements under the commercial name “CEA-06-125UN-350” (gage factor 2.085, resistance 350 Ohms, length 4.57 mm). Three strain gauges (Figure 10a) were fixed to the specimens with a bonded length of 150 mm and four strain gauges (Figure 10b) to the specimens with a 200-mm bonded length. Strain gauges were applied at a distance of 10 mm from the loaded end and with a mutual centre-to-centre distance of 50 mm (Figure 11).

Test results with the use of strain gauges were used to plot graphs in terms of strain *versus* location. The strain in the CFRP bar along the bonded length is plotted for different values of the load, indicated as a percentage of the failure load (10%, 20%, 50%, 75% and 100%, respectively). All points of the graphs were plotted from the strain gauge readings, with the exception of the strain at the unloaded end of the bars, which was assumed equal to zero and the loaded end (stress calculated from the axial load).

From the strain-location data, much useful information can be drawn. Strain distributions exhibit an approximately linear behaviour for low load levels. When the load increases, strain distribution along the different positions show an almost non-linear trend. This could mean that, as the axial load rises, the bond stresses become more evenly distributed along the bonded length as a consequence of variations in the characteristics of the bond. For low values of the axial load, the primary bond mechanism seems to be governed by the chemical adhesion due to the epoxy resin, but when the load increases, the primary bond mechanism changes from a chemical adhesion to mechanical friction mechanism between the bar’s indentation and the epoxy resin in the interface between the materials. This could be noted in Figure 8a: the cracks are parallel to the CFRP bar and particularly large near the bar indentations.

Strain readings obtained from strain gauges were used also to evaluate the axial stress σ_b_ values on the lateral surface of the CFRP bars using Hooke’s law:

(1) $${\text{σ}}_{\text{b}}=E_{\text{b}}\times {\text{ε}}_{\text{b}}$$

where *E*_b_ is Young’s modulus of the CFRP bar and ε_b_ the normal strain of the bar.

**Figure 12:**
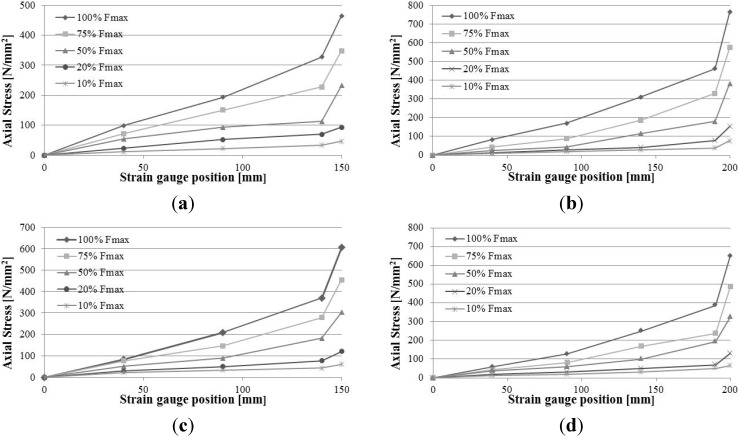
Normal stress *versus* position: (**a**) SF_2 test; (**b**) SF_4 test; (**c**) SC_2 test; (**d**) SC_4.

Results are reported in Figure 12. Due to the effect of the application of the load using the hydraulic jack, timber material was mainly in compression, and both CFRP bars epoxied on the notches were in tension. The distribution of the tensile axial stresses on the CFRP bars is linear near the unloaded end and parabolic at the loaded end. The maximum value of the tensile stress is always located near the loaded end. The non-linear behaviour is more evident for values of axial loads of 100%, 75% and 50% of the maximum load.

Data obtained from tests with the use of strain gauges finally were used to evaluate the bond stress τ_b_ between the CFRP bar and epoxy resin. The equilibrium of a CFRP bar’s element of length d*x*, with the hypothesis of linearly-elastic behaviour, is defined by the following:

(2) $${\text{τ}}_{\text{b}}(x)=\frac{d_{\text{b}}}{4}E_{\text{b}}\frac{{\text{dε}}_{\text{b}}(x)}{\text{d}x}$$

where: *d*_b_ = bar’s diameter; *E*_b_ = Young’s modulus of the bar; dε_b_ = strain of the bar element length d*x*. Because strain measurements are available at discrete points along the bonded length and indicating with ε_bi_ the strain reading at the location expressed by the coordinate *x*_i_ and with ε_bj_ the strain reading at the coordinate *x*_j_, Equation (2) could be approximated as:

(3) $${\text{τ}}_{\text{b}}(\frac{x_{\text{i}}+x_{\text{j}}}{2})=\frac{d_{\text{b}}}{4}E_{\text{b}}(\frac{{\text{ε}}_{\text{bj}}-{\text{ε}}_{\text{bi}}}{x_{\text{j}}-x_{\text{i}}})$$

**Figure 13:**
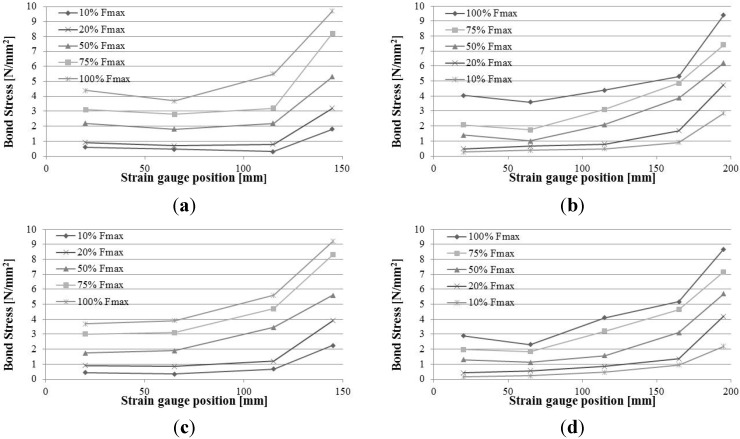
Bond stress *versus* position: (**a**) SF_2 test; (**b**) SF_4 test; (**c**) SC_2 test; (**d**) SC_4.

Experimental results obtained for the specimens equipped with strain gauges, using Equation (3), are shown in the following Figure 13. Due to the surface deformation of CFRP bars, the primary bond mechanism is the mechanical interlocking, while chemical adhesion of the epoxy resin is a secondary bond mechanism. During the tests, the radial components of the bond stresses generate micro-cracks in the epoxy resin and the consequent slip between bars and adhesive. This was observed for the SF_4, SC_2 and SC_3 tests. Micro-cracking and the consequent slip between the materials tend to cause the bond stress to be more evenly distributed. Figure 13 shows that, for low load levels, the bond stresses at the unloaded end is close to 0 N/mm^2^. As the load increases, the peak of the bond stress gradually shifts towards the loaded end, and it mainly contributes to resisting the external force applied by the jack.

### 4.3. Discussion and Comparison with Previous Research

Several bending tests carried out in the past by the same authors [15] have shown that the most frequent failure mechanism of CFRP-reinforced beams is due to cracking of timber on the tension side without an important plasticization of the timber in the compression side, depending on the quality of the wood. Wood yield is interrupted from beam failure due to the appearance of cracks, particularly for softwood (fir) beams.

Beams reinforced with CFRP bars revealed less ductile behaviour compared to that of those reinforced with CFRP sheets [15]. The CFRP reinforcement caused an increase of 52% for a double CFRP bar reinforcement. Load *vs*. deflection curves show that the beams exhibited a more basically linear behaviour up to failure. The positive effect induced by the presence of the bars does not suffice to confine local ruptures and bridge local defects. Moreover, the grooves cut into the beams in order to insert the CFRP bars produce some limited damage.

Results of this research on the bond strength of CFRP bars to timber show that an adequate stress transfer is possible both for soft and hardwood using limited bonded lengths (approximately 250 mm) and standard epoxy resins. However, the local character of the bar reinforcement is not able to prevent failure of the timber in the tension un-reinforced areas, especially at the beam edges. According to this, it could be advised that a “more uniform” reinforcement is to be preferred: numerous small FRP bars inserted in small notches produce a smaller damage and contribute to reducing stress and strain in the timber material.

## 5. Conclusions

This paper investigates the bond behaviour of NSM CFRP bars installed in softwood (fir) and hardwood (chestnut) timber. A series of 24 specimens, 12 in fir and 12 in chestnut wood, were tested to analyse the effect of different bonded lengths and different timber species on the bonded strength of the bars. The following conclusion can be drawn:

(1) The pull-out capacity in NSM CFRP bars at the onset of debonding increased with bonded length up to a length of 250 mm. The CFRP bar’s pull-out was achieved only for specimens with bonded lengths of 150 and 200 mm.
(2) The test results identified three basic modes of failure: one related to the parent material (*i.e*., timber); and the other two associated with the reinforcing composite material (CFRP bar pull-out and tensile failure, *i.e.*, rupture of CFRP bar or cracking of the epoxy system). For high bonded lengths (250 and 300 mm), timber failure was observed to be the controlling mode. For small bonded lengths (150 and 200 mm), either timber cracking or CFRP pull-out was observed, the latter being the most common.
(3) For 250- and 300-mm bonded lengths, rupture was always initiated by the failure of the carbon filaments, which are not parallel to the bar and produced a radial component of the bond stress. This component caused micro-cracks that slowly propagated up to determine a noticeable cracking of the epoxy resin and the subsequent slipping-out of the bar as the load increased.
(4) Timber type did not affect the bond behaviour: the different test results between fir wood (softwood) and chestnut wood (hardwood) specimens were small for all four different bonded lengths in terms of pull-out capacity and bond strength.

Further experimental investigation taking into account different bonded lengths, epoxy resins and types of FRP bars will be necessary to address the problem.
